# Performance Research and Structure Optimization of Labyrinth Screw Pump

**DOI:** 10.3390/mi12070790

**Published:** 2021-07-01

**Authors:** Xin Rong, Hongwu Zhu, Biao Hu

**Affiliations:** 1 College of Mechanical and Transportation Engineering, China University of Petroleum, Beijing 102249, China; zhuhw@cup.edu.cn; 2 Chongqing Changan Automobile Company Limited, Chongqing 401120, China; wxhubiao@126.com

**Keywords:** labyrinth screw pump, viscous oil transportation, response surface optimization, OSFD, sensitivity analysis, genetic algorithm

## Abstract

The labyrinth screw pump is a new type of low-flow rotor pump with a simple structure and good sealing performance. It is suitable for the transport of high-viscosity, high-gas-content, and particle-containing media. In this study, a rectangular labyrinth screw pump was used as the research object. The effect of the medium viscosity on the performance of the labyrinth pump was studied through numerical simulations, and the correctness of the simulation method was verified using existing test data. The efficiency and head of the labyrinth screw pump were selected as the optimization objectives, and the pump structural parameters were selected as the optimization parameters. A structural optimization model of the labyrinth screw pump based on response surface theory was established. The structural parameters of the labyrinth pump were reasonably simplified through size correlations, and then parameter sensitivity analysis was performed to determine the important structural parameters that needed to be optimized. The OSFD (optimized space-filling design) was used to combine the optimized parameters and generate the sample space. The response surface theory was combined with a neural network prediction model and a multi-objective genetic algorithm to perform optimization calculations. The results showed that there was an interactive influence between the structural parameters of the stator and rotor of the labyrinth screw pump. Compared with the original model, the optimized model pump had an efficiency increase of 13.55% and a lift increase of 19.53% when conveying a medium with a viscosity of 133 cp.

## 1. Introduction

As the viscosity of oil continues to increase and wellbore conditions continue to become more complicated, the lifespan and pump efficiencies of electric submersible pumps and screw pumps commonly used in oilfields have declined, affecting the normal operation of equipment, and the economic benefits have deteriorated year by year. At present, the most commonly used pumps in the exploitation of heavy oil are submersible electric pumps. However, the efficiencies of electric submersible pumps with semi-open impellers are lower than 30% when transporting liquids with viscosities greater than 50 cp, and pump leakage is significant [1,2]. The labyrinth screw pump (or labyrinth pump) is a non-contact power pump and a new type of screw pump with a small flow, high head, and low specific speed. It is suitable for the transport of high-viscosity, high-gas-content, and particle-containing media.

Many scholars have been committed to studying pump theory based on the labyrinth spiral seal mechanism. The earliest scientist who developed the labyrinth screw pump was a Soviet scientist. The purpose of the research and development at that time was to solve the problem of conveying viscous media containing particles. In the labyrinth spiral seal, Golubiev [3] found that the structure of opening a single or multiple threads on the surface of the ring seal could increase the pressure of the sealing liquid in the thread grooves, thereby achieving the purpose of preventing liquid leakage. Bilgen [4] and Karow [5] used laminar and turbulent flow models to numerically analyze the spiral structure of the labyrinth and obtained the flow characteristics in the spiral cavity of the labyrinth under different conditions. Zhu [6] used the laminar flow model to analyze the flow in the labyrinth spiral and obtained the plane flow solution and the spatial flow solution of the oblique section in the honeycomb body. In addition, some researchers have also conducted theoretical analysis and experimental research on the labyrinth seal structure [7,8,9]. These analysis results all showed that the labyrinth spiral structure can produce a greater pumping pressure at high speeds. In terms of the pumping mechanism, Golubiev [10] believed that the pumping pressure of the labyrinth screw pump was caused by the strong turbulent friction of the fluid between the rotor and the stator acting on the threaded wall. Bilgen and Akgungo [4] regarded the fluid flow in the pump as the superposition of the drag flow of the rotor thread on the fluid and the pressure flow under the pressure difference between the two ends of the pump. In terms of structural design, many researchers used computational fluid dynamics (CFD) to numerically simulate trapezoidal, triangular, and rectangular labyrinth pumps, respectively, and analyzed the influence of the thread design parameters on the pumping capacity [11,12,13]. Ma [14] calculated and compared the performances of labyrinth pumps with different thread shapes and found that the rectangular labyrinth screw pump is more suitable for transporting highly viscous media and for multiphase flows.

In the transportation of viscous media, the performance of the labyrinth screw pump is positively correlated to the viscosity of the media, and thus, it can be used in the petrochemical, pharmaceutical, metallurgical, and electric power industries. However, the efficiencies of the currently known labyrinth pumps are very low, which has limited their development. Some scholars have studied the advantages of labyrinth pumps in conveying air-containing, high-viscosity, and other media, but there have been few studies on the structural optimization of labyrinth screw pumps [15,16].

The optimization of a pump structure often depends on data samples collected through experimental design methods and uses various methods, such as Kriging or artificial neural network models, to construct the approximate functional relationship between the optimization parameters and the optimization objective. OPTIMUS, Tosca, and other platforms are used to estimate the functional relationships between the input parameters and output parameters through optimization models and algorithms, and the optimal control parameter combination can be obtained. However, in the process of hydraulic optimization, the optimization is still carried out with the help of design experience, and the optimization results are often not ideal. In contrast to the above optimization models, the essence of the response surface methodology is to replace the model with data. The response surface approach can estimate the variations of the entire design space based on the sample points obtained by the experimental design and graphically express the functional relationship between the input and output. The response surface provides estimated values for the output parameters, and the output function value can be obtained only through the response surface, without the need to perform operations on the original model. Therefore, using the response surface model to optimize the structure of the pump can reduce the calculation time considerably.

Zhang [17] selected a fluoroplastic two-phase-flow centrifugal pump as the research object, adopted the response surface optimization model, and optimized the structure of the pump with the main geometric parameters of the impeller as the optimization parameters to improve the efficiency of the pump and reduce the wear rate. Gao [18] determined the optimization parameters according to the degree of influence of the structural parameters on the objective function and selected the efficiency, shaft power, and head of the pump as the optimization objectives. A response surface optimization model between the structural parameters and the objective function was constructed, and the interactions between the structural parameters were examined.

The structure of the labyrinth screw pump is extremely complex, and the rotor and stator have different structural parameters. Furthermore, the fluid domain involves the handling of dynamic and static interfaces. As a result, the relationship between the objective function and the optimization parameters is difficult to express explicitly, and some optimization parameters are not continuous (the number of stator and rotor screw threads should be rounded according to actual engineering needs). Therefore, the traditional gradient optimization method is not suitable for this study. In response to these problems, we used response surface optimization technology to optimize the structure of the labyrinth pump. Selecting the fluid area of the main part of the rectangular labyrinth screw pump as the research object, the goal was to improve the efficiency and head of the pump and find the best combination of structural parameters.

Section 2 introduces the geometry and operation parameters of the labyrinth screw pump. Section 3 of this article presents the setup and experimental verification of the numerical simulation method. Section 4 describes the process of structural optimization. Section 5 discusses the influence of the internal flow and the oil viscosity on the performance of the labyrinth pump and analyzes the optimization results of the labyrinth pump. This article applies the neural network response surface optimization model to the labyrinth pump, which provides a certain theoretical reference for the design of the labyrinth pump.

## 2. Geometry of Labyrinth Screw Pump

**Figure 1:**
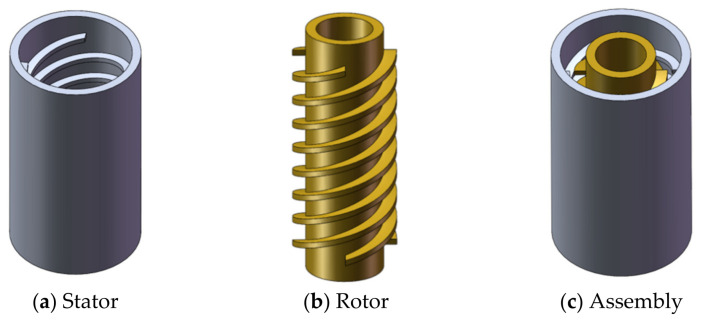
Structure and assembly of stator and rotor.

The main components of the labyrinth screw pump were a rotor and stator pair machined with opposite threads, and a radial clearance of 0.1–0.4 mm was left between the rotor and the stator. When operating, the rotation direction of the rotor was opposite to the direction of its own thread. The spiral groove of the labyrinth screw pump was very shallow, and the fluid produced a horizontal or longitudinal vortex in the stator and rotor grooves during the flow. A labyrinth pump mainly relies on the vortex in the groove to realize the momentum transfer of the fluid between the stator and the rotor [14]. Figure 1 shows a schematic diagram of the structure and assembly of the stator and rotor in the pump.

**Figure 2:**
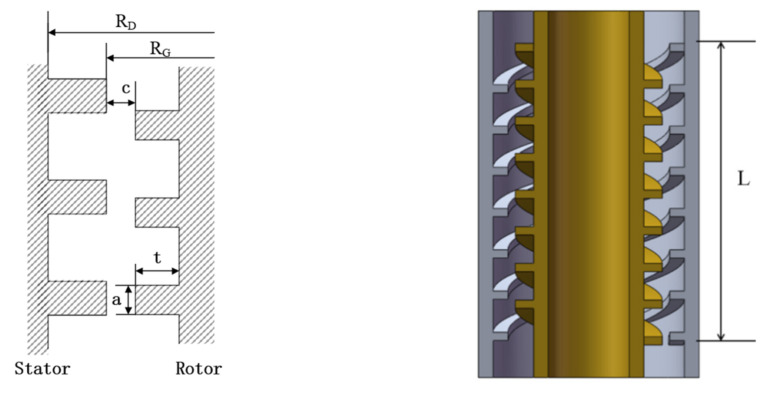
Description of the size parameters of the stator and rotor.

The operation parameters of the labyrinth pump studied were as follows: rotary speed *N* = 3000 rpm, volumetric flow rate *Q* = 4.17 m^3^/h, and hydraulic head *H* = 17 m. The geometric parameters of the rotor and stator are shown in Table 1. Figure 2 shows a schematic diagram of the main geometric parameters of the stator and rotor.

## 3. Steady-State Calculation of Fluid Domain of Labyrinth Screw Pump

### 3.1. Governing Equations

(1) Mass conservation equation:

(1) $$\frac{\partial \left(\rho u\right)}{\partial x}+\frac{\partial \left(\rho v\right)}{\partial y}+\frac{\partial \left(\rho w\right)}{\partial z}=0,$$

where *ρ* is the density of the fluid, and *u*, *v*, and *w* represent the coordinate components of the velocity.

(2) Momentum conservation equation:

(2) $$\frac{\partial \left(\rho u\right)}{\partial t}+div\left(\rho uu\right)=div\left(\mu gradu\right)-\frac{\partial p}{\partial x}+S_{u},\;\frac{\partial \left(\rho v\right)}{\partial t}+div\left(\rho vu\right)=div\left(\mu gradv\right)-\frac{\partial p}{\partial y}+S_{v},\;\frac{\partial \left(\rho w\right)}{\partial t}+div\left(\rho wu\right)=div\left(\mu gradw\right)-\frac{\partial p}{\partial z}+S_{w},$$

where *p* represents the pressure on the fluid micro-element body, *S*_u_, *S*_v_, and *S*_w_ represent the generalized source term in the momentum conservation equation, *µ* represents the dynamic viscosity, *u* represents the fluid absolute velocity vector, and *t* represents time.

(3) Energy conservation equation

The fluid calculation domain was set as an isothermal condition in this paper, so the energy conservation equation was omitted.

To solve the turbulence problem, a new turbulence model equation must be introduced to close the equations. The SST (shear stress transport) *k-ω* turbulence model was used due to its high precision and stability. The transport equations of the turbulent kinetic energy *k* and specific dissipation rate *ω* in the SST *k-ω* model are as follows:

(3) $$\frac{\partial }{\partial t}\left(\rho k\right)+\frac{\partial }{\partial x_{i}}\left(\rho ku_{i}\right)=\frac{\partial }{\partial x_{j}} [\left(\mu +\frac{\mu_{t}}{\sigma_{k}}\right)\frac{\partial k}{\partial x_{j}}]+G_{k}-Y_{k}+S_{k},\;\frac{\partial }{\partial t}\left(\rho \omega \right)+\frac{\partial }{\partial x_{i}}\left(\rho \omega u_{i}\right)=\frac{\partial }{\partial x_{j}} [\left(\mu +\frac{\mu_{t}}{\sigma_{\omega }}\right)\frac{\partial k}{\partial x_{j}}]+G_{\omega }-Y_{\omega }-D_{\omega }+S_{\omega },$$

where *G*_k_ and *G*_ω_ are the generation terms of turbulent kinetic energy *k* and dissipation rate *ω* caused by the average velocity, respectively; *σ*_k_ and *σ*_ω_ are the turbulent Prandtl numbers of *k* and *ω*, respectively; *Y*_k_ and *Y*_ω_ are turbulence caused by *k* and *ω*, respectively; *D*_ω_ is the cross-diffusion term; and *S*_k_ and *S*_ω_ are user-defined source terms.

### 3.2. Mesh Division and Mesh Verification

**Figure 3:**
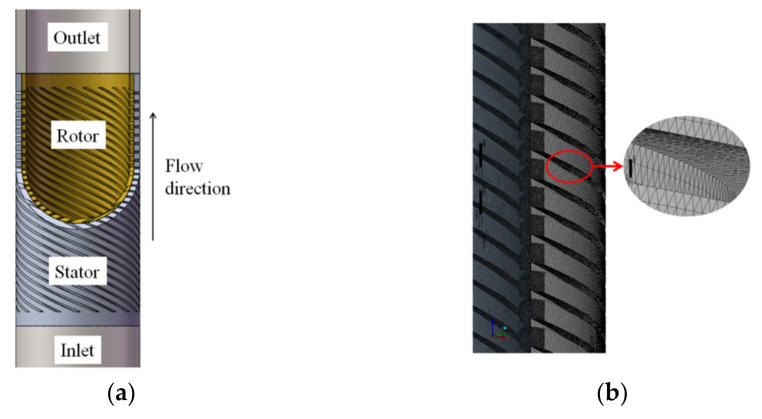
(**a**) Model of stator and rotor fluid domain and (**b**) partial mesh section view.

**Figure 4:**
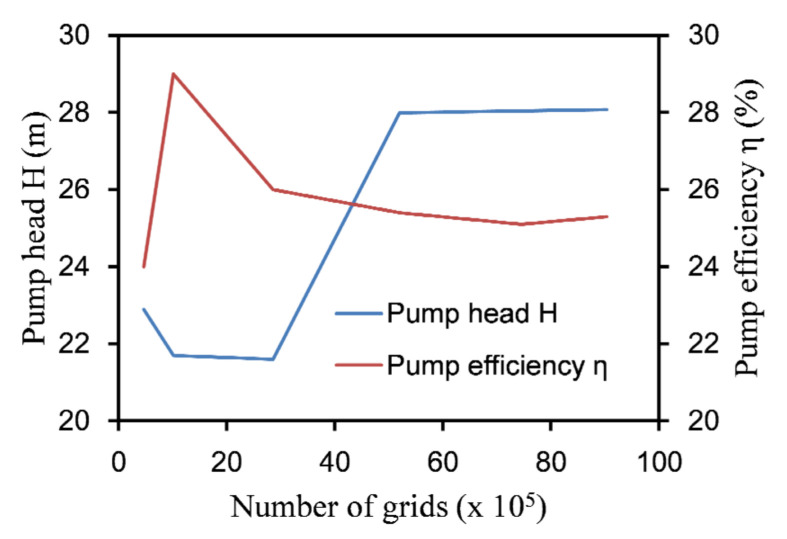
Grid independence verification.

The UG software (UG is a CAD/CAM/CAE software system produced by Siemens PLM Software, headquartered in Plano, Texas, USA) was used to model the inlet and outlet sections as well as the rotor and stator of the labyrinth pump in three dimensions. To develop the turbulence in the pipe fully, the inlet and outlet sections were extended to five times the pipe diameter. To refine the grid at the gap between the stator and rotor, the relevance at the contact region was set to the maximum value of 100. Figure 3 shows a mesh cross-sectional view of the stator and rotor parts. To determine the appropriate number of grids, the grid independence of the computational domain was verified, and the result is shown in Figure 4. The calculation formulas of the labyrinth pump head H and the efficiency η are as follows [19]:

(4) $$H=\frac{P_{\mathrm{outlet}}-P_{\mathrm{inlet}}}{\rho g}+(z_{\mathrm{outlet}}-z_{\mathrm{inlet}}),$$

(5) $$P_{\mathrm{zhou}}=\frac{\pi Tn}{30},$$

(6) $$\eta =\frac{\rho gQH}{P_{\mathrm{zhou}}}\times 100\%,$$

where *P*_outlet_ and *P*_inlet_ are the total pressure at the outlet and inlet, respectively (Pa); *z*_outlet_ and *z*_inlet_ are the height at the inlet and outlet, respectively (m); *P*_zhou_ is the shaft power (W); *T* is the input torque (N·m); *n* is the speed (r/min); *Q* is the volume flow (m^3^/s); *ρ* is the density of the conveying medium (kg/m^3^); and *g* is the acceleration of gravity (m/s^2^).

Due to the special structure of the labyrinth screw pump, the pump head and efficiency have a close relationship with the size of the stator and rotor gap. The finer the grid is, the more it can capture the influence of the stator and rotor gap on the pump performance. Therefore, when the resolution of the grid increases, the lift and efficiency of the model pump will fluctuate. Figure 4 shows that when the total number of grid cells was 5,198,481, the pump lift and efficiency tended to be stable. At this time, the numbers of grid cells of the inlet section, outlet section, rotor, and stator were 514,273, 518,554, 2,501,373, and 1,664,281, respectively.

### 3.3. Model Settings and Boundary Conditions

With flow inlet and static pressure outlet, no-slip mode was imposed on the wall. Because there were interactions between the stator, rotor, inlet, and outlet, and the number of stators and rotors was large, it could not be set as a periodic interface. The rotor domain was set as a rotating reference system, and the stator domain was set as a fixed domain. The general grid interface (GGI) method in General Connection was used to realize the data coupling between various computing domains. This method can establish a secure and stable connection at the interface, even though the grid nodes on both sides of the interface were inconsistent. The Frozen Rotor method was selected for the connection between the rotor domain and the inlet section, outlet section, and stator domain. This method ignored the transient influence on the interface when the reference coordinate system changed, which could reduce the number of calculations and the computational cost.

### 3.4. Test Validation

To verify the correctness of the numerical simulation method, it was compared with the experimental data of the rectangular labyrinth screw pump of Jiangsu University [20,21]. The speed was 3000 rpm. The physical properties of the medium were water and oil. At room temperature, the density of water was 997 kg/m^3^, the viscosity was 1 cp, the density of oil was 1203 kg/m^3^, and the viscosity was 53 cp.

**Figure 5:**
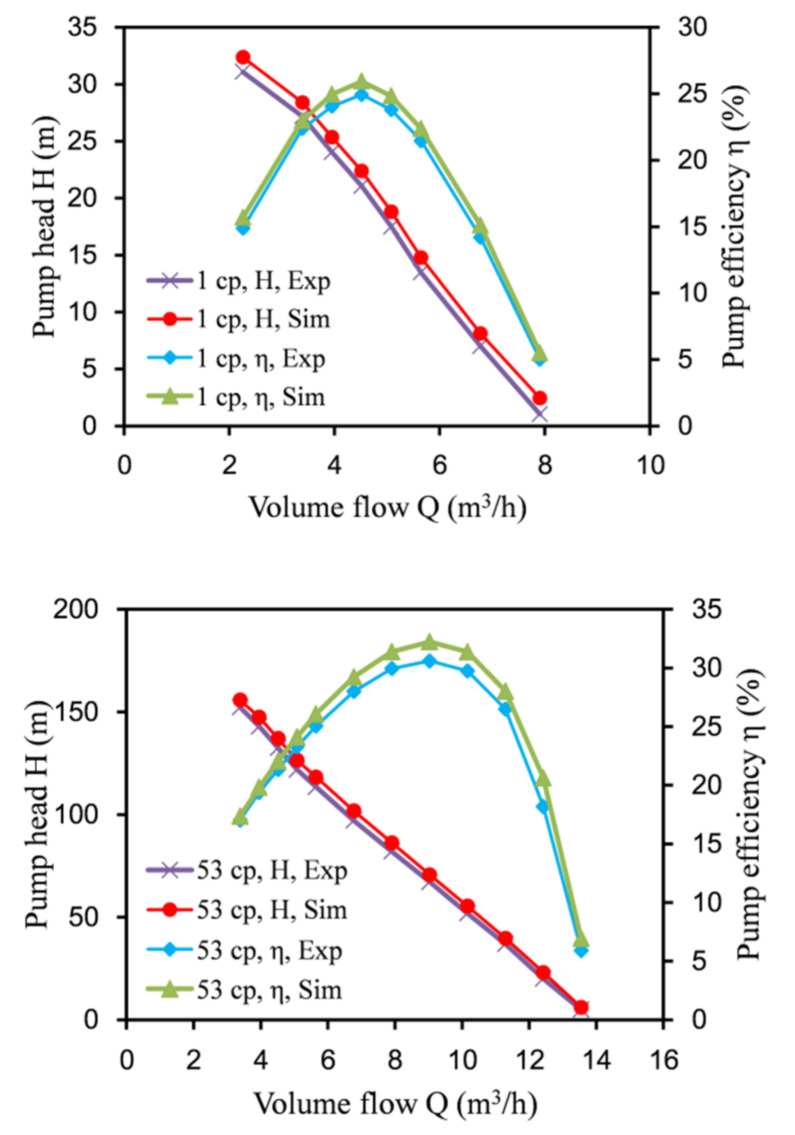
Comparison of test and simulation results.

Figure 5 shows the comparison of the test and simulation results of the rectangular labyrinth screw pump. The numerical calculation results were more consistent with the experimental data. After the calculation, when the medium was pure water or viscous oil, the simulation error of the lift and efficiency could be controlled to within 5%. This showed that the numerical calculation method was reliable, and the performance of the labyrinth screw pump could be predicted through the numerical simulation.

## 4. Structure Optimization

**Figure 6:**
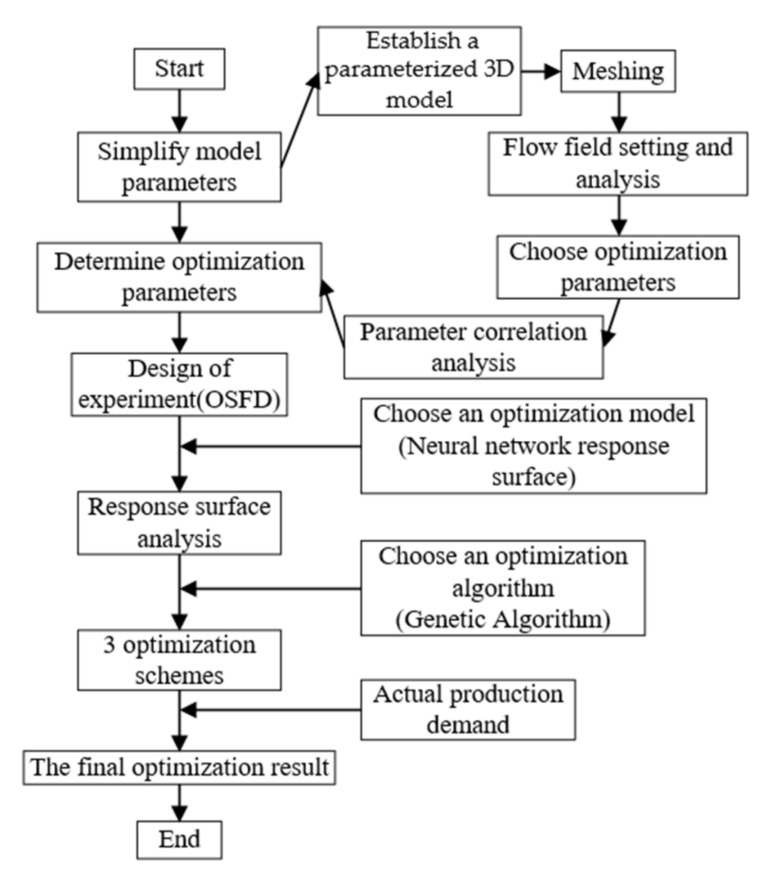
Structure optimization process.

The head *H* of the labyrinth pump and the efficiency *η* were selected as the optimization targets of the response surface optimization. The specific process is shown in Figure 6. First, a parameterized three-dimensional model of the spiral labyrinth pump was established in UG. The structural parameters of the model pump were identified and controlled through the parameterization function of the ANSYS Workbench, and the target function that needed to be output in the fluid calculation module was set to establish correspondence between the structural parameters and the objective function. An optimal space-filling design (OSFD) was used to determine the required test points. The test points were calculated one by one under the response surface module, and the structural parameters were fitted in the response surface optimization module using the obtained test data. The mathematical model relating the efficiency and head was an optimization model based on the response surface. On this basis, the multi-objective genetic optimization algorithm was used to obtain a series of solutions that met the requirements based on the model, and one of the solutions was selected as the final structural optimization size according to the actual engineering needs [22,23,24].

### 4.1. Parameter Correlation Analysis and Optimized Space-Filling Design

The structure of the rotor and the stator involved a total of 16 structural design parameters, and the parameters could have interactive effects. Based on the correlation between the model sizes, the number of design parameters could be simplified to 11. To keep the helix angle constant, the ratio of the stator inner diameter *d*_ns_ to the stator lead *s*_s_ was set to k_1_, the ratio of the rotor outer diameter *d*_nr_ to the rotor lead *s*_r_ was set to *k*_2_, and the ratio of the rotor tooth depth *t*_r_ to the stator tooth depth *t*_s_ was set to *k*_3_. Table 2 shows the simplified structural design parameters.

To exclude the parameters that had little influence on the external characteristics of the labyrinth screw pump, sensitivity analysis of the parameters was carried out to select the design parameters that needed to be optimized. The Parameters Correlation module of the ANSYS Workbench (ANSYS, 2020R1, ANSYS, Inc., Pittsburgh, PA, USA) was used to obtain the sensitivity distribution of each design parameter to the optimization target. The module uses the Spearman correlation coefficient method to analyze the parameter sensitivity. This method can be used to measure the strength of the connection between the input and output parameters. The larger the absolute value is, the greater the influence of the input parameter is on the output parameter. A positive value indicates that the output parameter is positively correlated with the input parameter. The correlation coefficient of this method is defined as follows [25]:

(7) $$P_{s}=\frac{\sum_{i=1}^{N}\left(R_{i}-\tilde{R})(S_{i}-\tilde{S}\right)}{\sqrt{\sum_{i=1}^{N}(R_{i}-\tilde{R}{)}^{2}\sum_{i=1}^{N}{\left(S_{i}-\tilde{S}\right)}^{2}}}=1-\frac{6\sum d_{i}{}^{2}}{N(N^{2}-1)},$$

where $R_{i}$ and $S_{i}$ are the levels of observation *i*; $\tilde{R}$ and $\tilde{S}$ are the average levels of the variables *x* and *y*, respectively; and *N* is the total number of observations. $d_{i}$ = $R_{i}$ − $S_{i}$ represents the level difference of two pairs of variables.

There are only two factors that affect the accuracy of the response surface model: (1) the number and location of the sample points, and (2) the type of response surface. Therefore, the selection of sample points in the construction of the response surface is critical. There are many ways to generate sample points. The most common sampling method is the Latin hypercube sampling design (LHSD), and the optimal space-filling design (OSFD) is optimized based on the LHSD. The OSFD has better space-filling capabilities than the LHSD and is suitable for generating more complex response surfaces. Therefore, we used the OSFD method to generate the sample points [26].

### 4.2. Neural Network Response Surface

The results of the numerical experiments were used to generate response surfaces. The response variables in this analysis were the pump head and efficiency. In this case, there was a highly non-linear relationship between the input parameters, so a neural-network-type response surface was selected for modeling. The outstanding advantages of this method are its robustness and adaptability, that is, when there are too many input parameters or the response is noisy, it can automatically extract features that are useful for problem solving based on the samples to obtain better results.

The neural network prediction model is a mathematical model that abstracts the neural network of the human brain from the perspective of information processing. The neural network is composed of interconnected computing units (that is, neurons). Each neuron will be calculated at the node. Each neuron connection has an associated weight coefficient. After obtaining the predicted value of the output node of the network and the respective prediction error, the weight coefficients in the network will be updated to reduce the prediction error of the model. This iterative process will continue to repeat until the model error meets the convergence criterion.

### 4.3. Genetic Algorithm Optimization

According to the determined optimization parameters and the established response surface model, a multi-objective optimization method based on the genetic algorithm was used to optimize the structure of the spiral labyrinth pump. A multi-objective genetic algorithm can find global and local optimal values, and it can find multiple candidate points in different design areas, instead of the only global optimal solution. Therefore, after obtaining the optimal solution set by the multi-objective genetic algorithm, it is necessary to find as many optimal solutions as possible that meet the optimization conditions and select one of them as the final result of the optimization based on the actual needs.

## 5. Results and Discussion

### 5.1. Analysis of Flow and Pressure Fields

#### 5.1.1. Velocity Distribution

**Figure 7:**
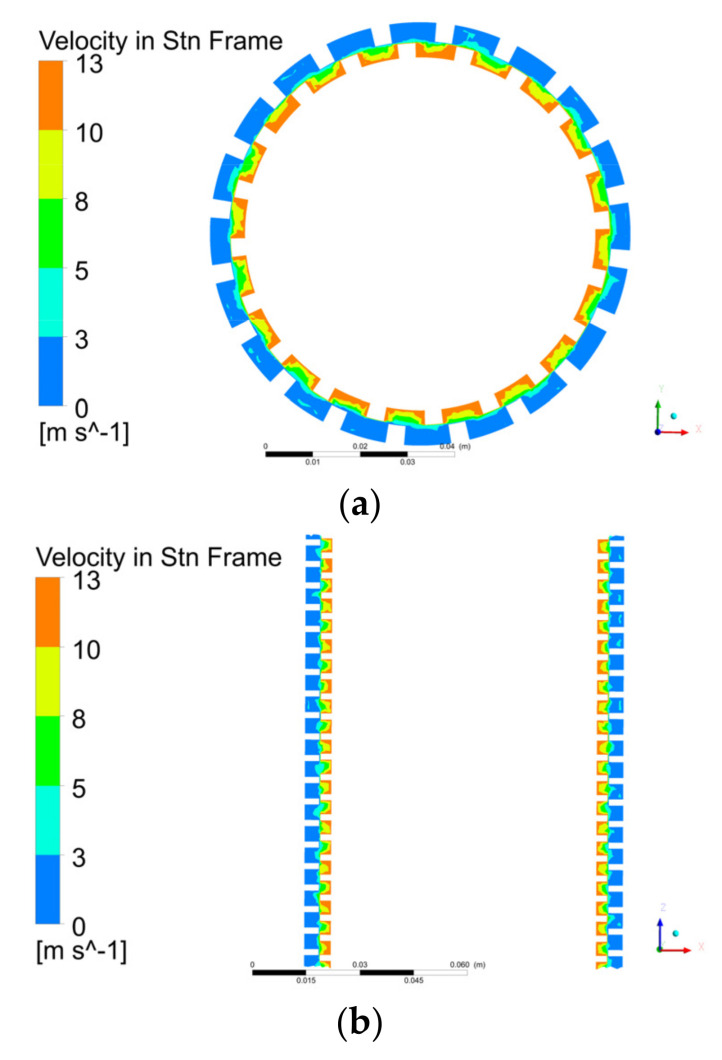
Contours of fluid speed distribution in the pump on the (**a**) XY and (**b**) XZ planes.

Figure 7 shows that the fluid velocity in the rotor was greater than the fluid velocity in the stator, the velocity of the fluid in the rotor changed more drastically than that in the stator, and the velocity distribution on the XY and XZ planes were the same for each stator and rotor pair. Therefore, by analyzing the velocity distribution of a stator and rotor pair, the overall flow in the pump can be understood.

(1) Radial velocity distribution in spiral unit

**Figure 8:**
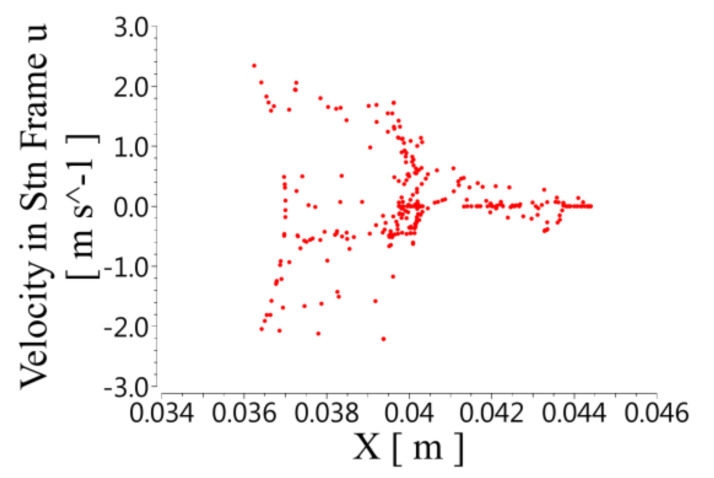
Distribution of radial velocity at different X positions on the XY plane.

Figure 8 shows the radial velocity distribution. The positive values of the speed correspond to centrifugal motion, and the negative values correspond to centripetal motion. The centrifugal and centripetal motion occurred in both the rotor and the stator, so the fluid in the stator and the rotor formed vortices in the radial direction. The radial velocity was unevenly distributed along the axial direction. The speed was highest when it was close to the wall of the rotor and gradually decreased with the increase in the radius. The radial velocity at the bottom of the stator slot was close to 0.

(2) Circumferential velocity distribution in spiral unit

**Figure 9:**
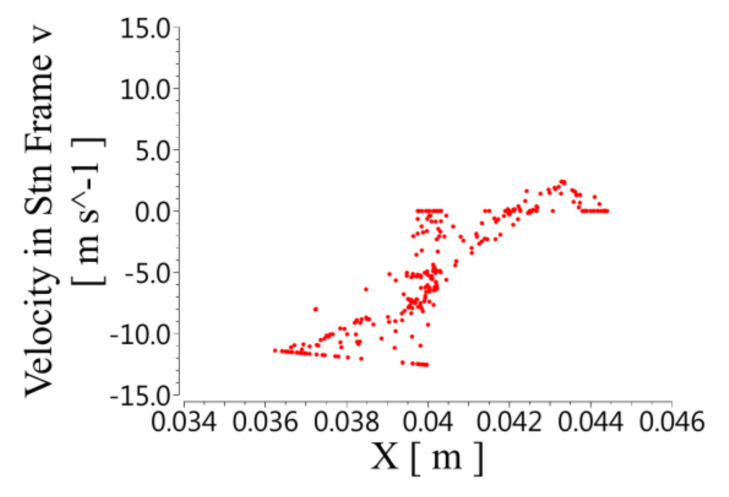
Distribution of circumferential velocity at different X positions on the XY plane.

Figure 9 shows the circumferential velocity distribution on the XY plane. The negative circumferential speed was the same as the rotor rotation direction. In the same section, the circumferential velocity of the fluid at the rotor and the gap was the same as the direction of rotation of the rotor, and vortices were formed in the stator. The circumferential speed was highest at the root of the rotor and gradually decreased as the radius increased. In the stator, as the radius increased, when the negative circumferential velocity gradually decreased to 0, a positive circumferential velocity gradually increased. When the magnitude of the positive circumferential velocity increased to a certain value, the forward velocity gradually decreased. In the rotor, the closer the fluid to the wall, the greater its velocity. In the stator, the reverse speed was greater when closer to the central area, and there was no circumferential speed at the wall.

(3) Axial velocity distribution in spiral unit

**Figure 10:**
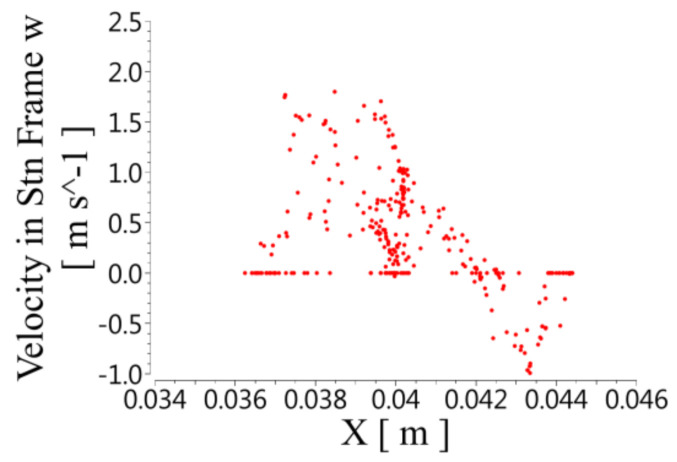
Distribution of axial velocity at different X positions on the XY plane.

Figure 10 shows the axial velocity distribution on the XY plane. The axial velocity in the rotor and the intermediate gap was consistent with the forward direction of the fluid, but there was a reverse flow in the stator. The axial speed was highest in the rotor, and the speed gradually decreased as the radius increased. In the stator, as the radius increased, the positive axial speed in the stator gradually decreased to 0. As the radius increased further, a negative axial velocity appeared, and the magnitude of this reverse velocity continuously increased. When it reached a certain value, the magnitude of this reverse velocity began to decrease as the radius further increased.

#### 5.1.2. Pressure Distribution in Pump

**Figure 11:**
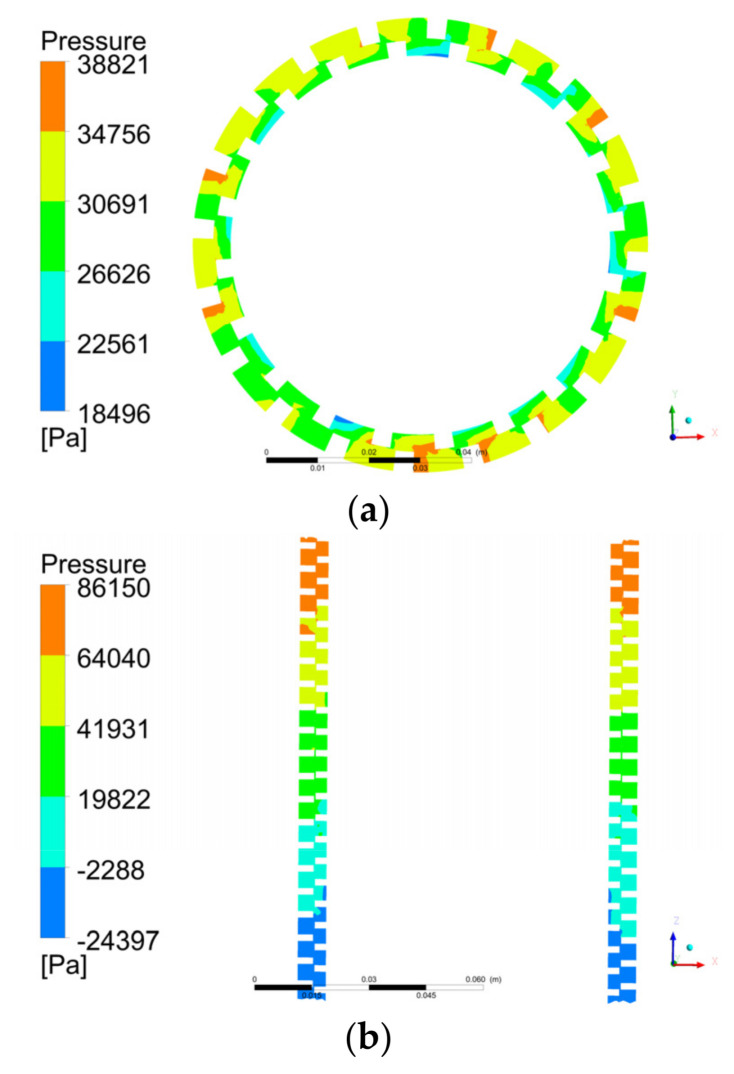
Contours of pressure distribution in the pump on the (**a**) XY and (**b**) XZ planes.

Figure 11a shows the pressure distribution in the pump on the XY plane. The liquid pressure gradually increased in the radial direction. The liquid pressure was highest at the bottom of the stator and lowest at the bottom of the rotor. This was because the oil obtained a higher kinetic energy in the high-speed rotating rotor. When the fluid entered the stator from the rotor, it was blocked by the stationary wall of the stator, and part of the kinetic energy was converted into pressure energy. Therefore, the fluid pressure in the stator was higher than that in the rotor. This was consistent with the conclusion obtained from the velocity analysis in Section 5.1.1, that is, the fluid velocity in the low-pressure area was relatively high.

Figure 11b shows the pressure distribution in the pump on the XZ plane. The pressure of the fluid increased linearly along the axial direction and reached the maximum value at the outlet of the spiral section, which also showed that the pump had a pressurizing effect on the fluid.

The obtained flow field characteristics are the same as Li’s conclusion [27], which verifies that the simulation results in this study are accurate.

#### 5.1.3. Pumping Mechanism

Based on the flow field analysis, we analyzed the motion of the fluid in the labyrinth pump and the pumping mechanism. When the rotor rotated, the pressure and kinetic energy of the fluid increased under the action of the pressure surface of the rotor, and a strong centrifugal movement around the pressure surface of the rotor was produced, causing the fluid to enter the stator from the rotor. The fluid in the stator was blocked by the static wall, and part of the kinetic energy was converted into pressure energy. At the same time, under the action of the pressure difference between the stator pressure surface and the rotor suction surface, part of the fluid in the stator flowed back to the rotor in the radial direction, and again, the fluid obtained higher kinetic energy in the rotor. Furthermore, the flow velocity in the circumferential direction was the main component; the axial velocity was small, and there were many longitudinal and radial vortices in the pump.

**Figure 12:**
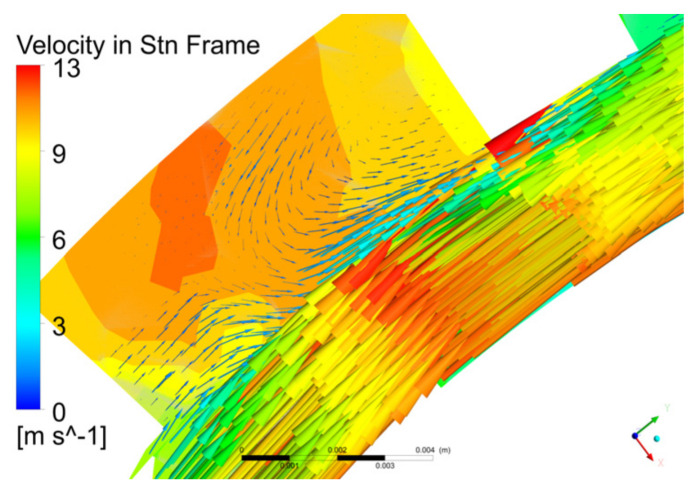
Changes in flow velocity at different pressures on the XY plane.

The spiral groove of the labyrinth pump was very shallow, and the centrifugal force was small. Therefore, the labyrinth pump mainly relied on the turbulent shear force of the fluid in the rotor and stator to increase the radial pressure difference between the rotor pressure surface and the stator suction surface. This allowed the fluid to generate a radial flow from the rotor to the stator. Furthermore, as shown in Figure 12, part of the fluid in the stator flowed back into the rotor due to the radial pressure difference between the stator pressure surface and the rotor suction surface, thereby forming a radial vortex in the stator. Because of the radial vortex, the fluid in the labyrinth pump periodically transferred the momentum of the fluid in the rotor to the fluid in the stator and then repeatedly entered the rotor to obtain energy. Therefore, the labyrinth pump mainly relied on these vortices to realize the momentum transfer between the fluid in the rotor and the fluid in the stator. The frequent energy conversion of the liquid in the pump led to an increase in the pump head, but it also produced a large amount of energy loss. Therefore, the flow and head curve of the labyrinth pump was steep, and the pump efficiency was low.

### 5.2. Influence of Oil Viscosity on Performance of Labyrinth Screw Pump

**Figure 13:**
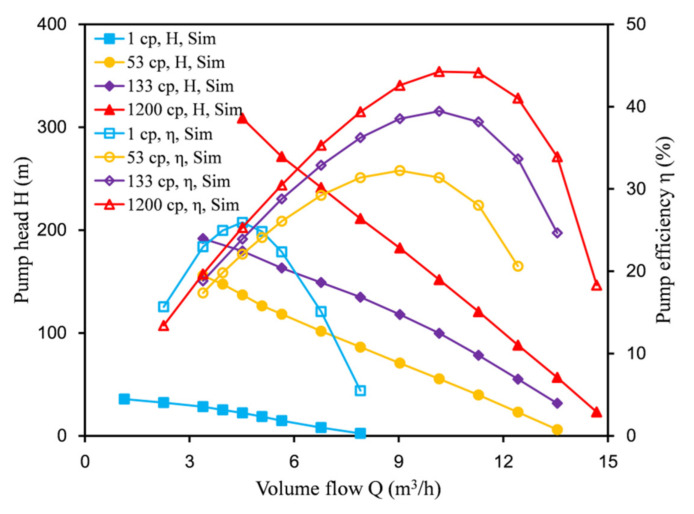
Influence of medium viscosity on the performance of the labyrinth pump.

Figure 13 shows the change of the head and efficiency with the flow rate at the same speed (3000 rpm) and different viscosities. With the increase in the oil viscosity, the head and efficiency of the labyrinth pump showed an upward trend. The best efficiency point of the pump moved to the right, and the liquid flow rate that could be delivered increased. This feature is different from centrifugal pumps. This is because the labyrinth pump reduces internal leakage when transporting high-viscosity media, thereby boosting the performance of the pump and increasing the head.

**Figure 14:**
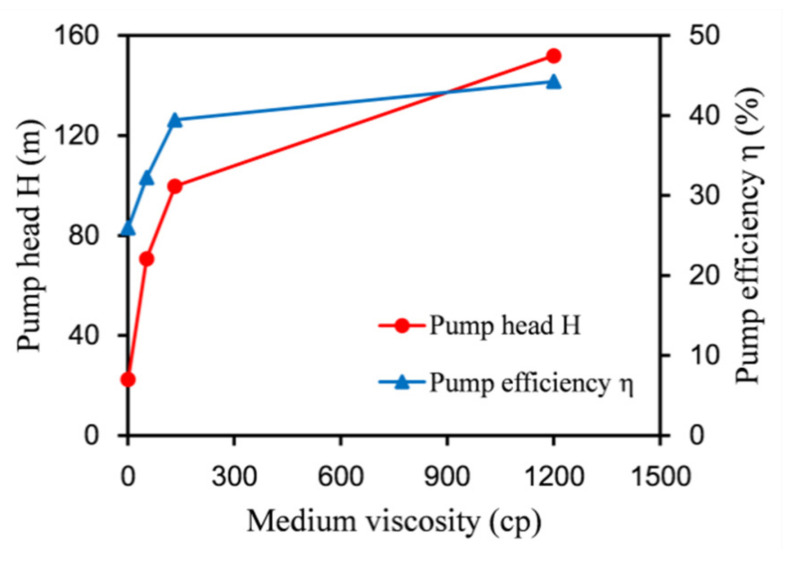
Performance comparison of labyrinth pumps at the best working point under different viscosities.

Figure 14 shows the variation of the head and efficiency with viscosity at the best efficiency point. When the oil viscosity was less than 133 cp, the efficiency increased faster, from 25.94% for 1 cp to 39.45% for 133 cp, corresponding to an increase of 52.1%. When the viscosity of the medium continued to increase, the increase in the efficiency dropped sharply. The efficiency increased from 39.45% for 133 cp to 44.26% for 1200 cp, corresponding to an increase of only 10.87%. When the viscosity continued to increase, the efficiency gradually leveled off. The variation trend of the head was the same as the efficiency. After the optimization, the head increased faster when it was less than 133 cp, from 27.14 m for 1 cp to 119.21 m for 133 cp, corresponding to an increase of 339.24%. When the viscosity continued to increase, the growth rate of the head decreased. The head increased from 119.21 m for 133 cp to 168.36 m for 1200 cp, corresponding to an increase of only 41.23%. All of these results indicate that the labyrinth screw pump is more suitable for conveying viscous media than clean water.

### 5.3. Structure Optimization

#### 5.3.1. Parameter Correlation Analysis and Sample Space

**Figure 15:**
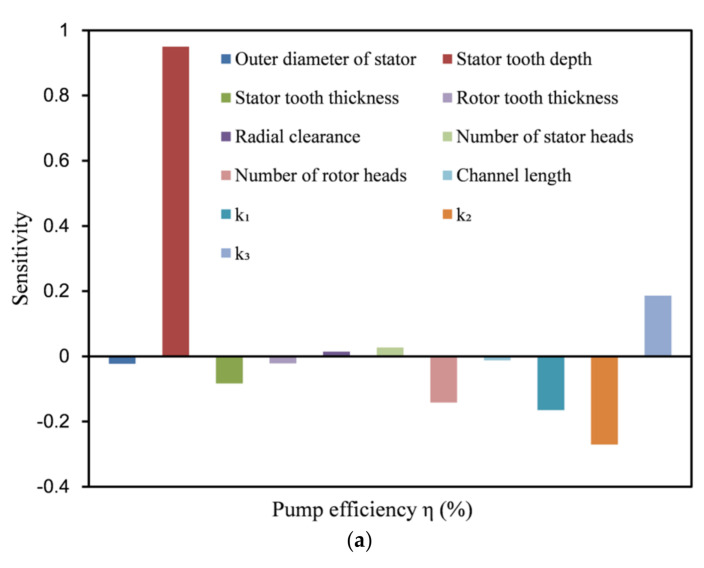

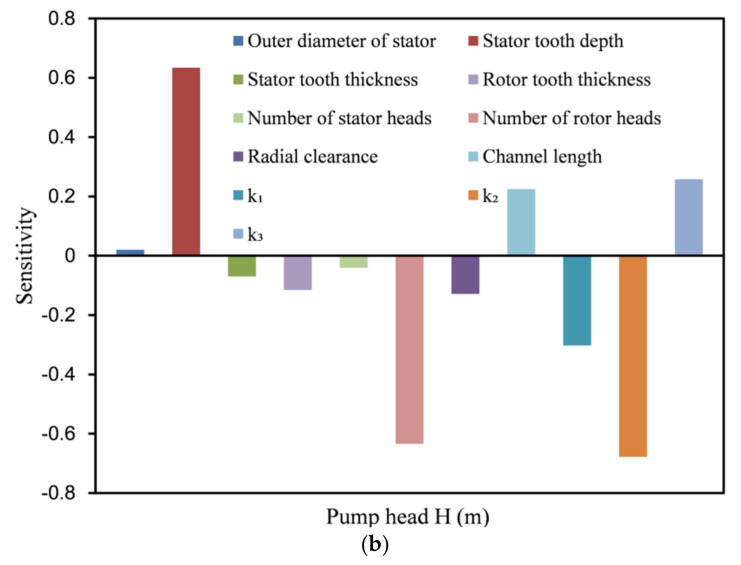
Sensitivity analysis results of design parameters for the (**a**) labyrinth pump head and (**b**) efficiency.

Figure 15 shows the sensitivity of the head and efficiency of the labyrinth pump to each design parameter from the sensitivity analysis. Figure 15a,b show that the pump head and efficiency were most sensitive to the depth of the stator teeth *t*_s_, while the efficiency had a very low sensitivity to the spiral body length *L*, only −0.012. Therefore, the influence of the length *L* of the spiral body on the efficiency of the labyrinth screw pump could be ignored. The sensitivities of the efficiency and head of the model pump to the stator outer diameter *d*_ws_ were relatively low, only −0.021 and 0.019, respectively. As this research focused on improving the efficiency of the model pump, the stator outer diameter *d*_ws_ and the spiral body length *L* were set as fixed values in the subsequent optimization process (the initial value of the outer diameter of the stator was 88.8 mm, and the length of the spiral body was the initial value of 160 mm) and no longer participated in the optimization process, reducing the optimization calculation time.

Based on the nine input parameters, ANSYS used the OSFD method to automatically generate 283 numerical experiments. The distribution of test points in the design space and the calculation results are shown in Table 3.

#### 5.3.2. Response Surface Analysis

Based on the response surface results, the effects of combinations of different input parameters on the objective function were examined, and the value ranges of the optimization results were determined according to the response surfaces [28]. Due to the excessive number of input parameters in this study, the influences of the interactions between the stator tooth depth *t*_s_, the number of rotor heads *z*_r_, and *k*_3_ on the model pump head and efficiency are presented as an example.

**Figure 16:**
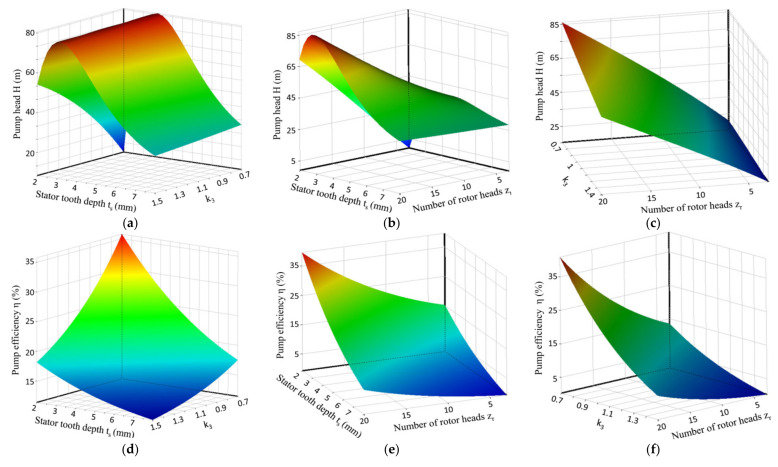
Response surfaces showing the effects of the interactions between the stator tooth depth *t*_s_, the number of rotor heads *z*_r_, and *k*_3_ on the pump head and efficiency of the labyrinth screw pump; (**a**,**d**) show the influence of *t*_s_ and *k*_3_ on pump head and efficiency; (**b**,**e**) show the influence of ts and zr on pump head and efficiency; (**c**,**f**) show the influence of *k*_3_ and *z*_r_ on pump head and efficiency.

Figure 16 presents the response surfaces, showing the influences of the stator tooth depth *t*_s_, the number of rotor heads *z*_r_, and *k*_3_ on the head and efficiency. Figure 16a,d show the influence of *t*_s_ and *k*_3_ on pump head and efficiency, respectively. It can be seen that the smaller the stator tooth depth and *k*_3_ were, the greater the efficiency, but for the head, regardless of the value of *k*_3_, the head first increased and then decreased with the stator tooth depth. Therefore, the value of *k*_3_ should be selected from the range of 0.7–1.1. Figure 16b,e show the influence of *t*_s_ and *z*_r_ on pump head and efficiency, respectively. As shown in Figure 16b,e, regardless of how the number of rotor heads changed, the efficiency decreased with the increase in the stator tooth depth, and the head first increased and then decreased. In the stator, the pump head could reach the maximum value when the tooth depth was 3, so the stator tooth depth *t*_s_ should be within the range of 2.0–4.0. Figure 16c,f show the influence of *k*_3_ and *z*_r_ on pump head and efficiency, respectively. Figure 16c,f show that regardless of how *k*_3_ changes, the head and efficiency increase with the increase in the number of rotor heads. Therefore, the number of rotor heads *z*_r_ should be in the range of 15–20. In the selection of parameters, the number of stator heads *z*_s_ and the number of rotor heads *z*_r_ need to be rounded according to engineering conditions.

#### 5.3.3. Analysis of Optimization Results

Table 4 shows the comparison of the three optimization schemes in the solution set with an efficiency greater than 35%. These three schemes greatly improved the efficiency and lift of the pump. Based on a comprehensive examination of the head and efficiency, plan 2 was selected as the final optimization result to generate the model. Table 5 shows the comparison of the specific structural parameters of the labyrinth screw pump before and after optimization.

Three-dimensional modeling and numerical calculations were carried out using the design parameters obtained by the optimization. Since the stator and rotor gaps changed, the grid independence verification needed to be performed again. The steps were the same as those in Section 3.2. The boundary design conditions in the pre-processing remained unchanged.

**Figure 17:**
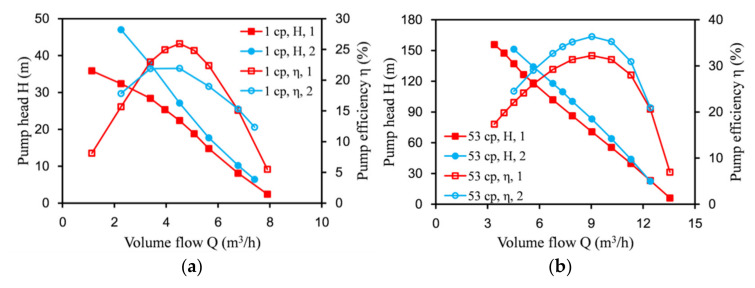

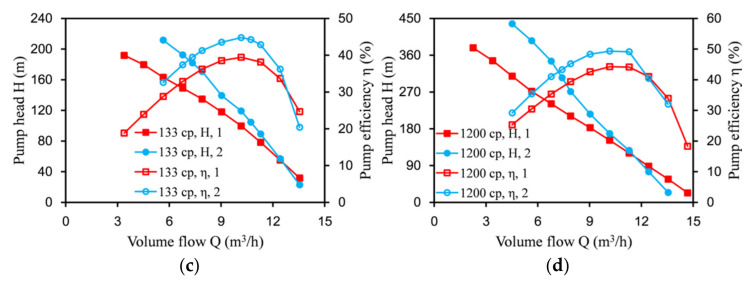
(**a**–**d**) show the performance comparison between (1) the original model and (2) the optimized model when the viscosity of transporting media is 1 cP, 54 cP, 133 cP and 1200 cP.

Figure 17a–d show the performance comparison between (1) the original model and (2) the optimized model when the viscosity of transporting media is 1cP, 54cP, 133cP and 1200cP, respectively. The increase in the drop speed of the head of the optimized labyrinth pump resulted in a smaller effective working area of the labyrinth pump, but the structural optimization did not change the flow at the best operating point. Table 6 shows the comparison of the target values at the best operating point before and after optimization. In addition to the 7.77% drop in efficiency of the optimized model pump under pure water conditions, the lift and efficiency increased by more than 10% when transporting viscous media, and the pumping performance of the labyrinth screw pump was significantly improved. Since this article focuses more on the optimization of the performance of the pump when conveying viscous media, the optimization presented above was considered to be successful.

## 6. Conclusions and Future Work

### 6.1. Conclusions

In this paper, a rectangular labyrinth screw pump was selected to study the influence of the viscosity of the conveying medium on the performance of the pump. Based on the response surface method, the main structural parameters of the stator and rotor were optimized. The main conclusions of this article are as follows:

(1) The performances of rectangular labyrinth pumps when conveying viscous oil are better than those when conveying water. As the viscosity of the conveying medium increases, the lift and efficiency of the pump will increase. This is because the increase in the viscosity of the medium reduces the leakage of the pump during the conveying process and reduces the energy loss.
(2) Based on the mathematical model of the pump performance parameters obtained by the response surface method, the calculation results are similar to the test results. Therefore, the optimization design method of the labyrinth screw pump based on the response surface method is reliable.
(3) After optimization, when the viscosity of the medium varied, the efficiency and head under each flow condition were significantly improved, except that the efficiency of pure water was reduced by 7.77%, and the head and efficiency for the other viscosities were increased by more than 10%.
(4) The surface response method can truly reflect the highly non-linear relationship and interactive influence between the design variables of the main structure of the labyrinth screw pump and the optimization goal. Furthermore, it provides an intuitive, efficient, and reliable optimization method for the control of the booster performance and efficiency of the labyrinth screw pump method.
(5) When the speed was 3000 rpm and the viscosity of the conveying medium was 133 cp, the efficiency of the optimized labyrinth screw pump reached 44.79%. This performance is higher than those of electric submersible pumps under the same working conditions. This shows that the labyrinth screw pump has the ability to handle high-viscosity liquids and exhibit better performances than electric submersible pumps when transporting viscous media.

### 6.2. Future Work

(1) When the viscosity of the conveying medium increases, the performance change of the labyrinth pump is opposite to that of the centrifugal pump. At present, the performance changes of pumps can only be obtained based on test data or simulation results, and there is no definitive theory on the pumping mechanism of labyrinth pumps.
(2) Since the radial gap between the stator and rotor of the labyrinth pump is small, the machining accuracy and materials of the stator and rotor will have certain requirements. Thus, we can study the processing costs and process issues.
(3) In actual production, the temperature of the liquid will change when the viscous medium is transported. However, in the simulation in this article, to simplify the calculation, we did not consider the temperature changes. Therefore, the influence of temperature on the performance of the labyrinth pump can be studied.

## Figures and Tables

**Table 1:** Geometric parameters of model pump.

Structural Parameters of Pump	Symbol	Rotor	Stator
Tooth depth (mm)	*t*	3	4
Number of threads	*z*	20	20
Addendum circle radius (mm)	*R*_D_	40	44.4
Root radius (mm)	*R*_G_	37	40.4
Tooth thickness (mm)	*a*	1.7	1.7
Lead of thread (mm)	*s*	112	120
Channel length of components (mm)	*L*	160	
Radial clearance (mm)	*c*	0.4	

**Table 2:** Design parameters and value range.

Design Parameters	Parameter Description	Initial Value (mm)	Optimization Range (mm)
*d*_ws_	Outer diameter of stator	88.8	88–91
*t*_s_	Stator tooth depth	4	2–8
*a*_s_	Stator tooth thickness	1.7	0.5–2.0
*c*	Radial clearance	0.4	0.5–1.5
*a*_r_	Rotor tooth thickness	1.7	0.5–2.5
*k*_1_	*k*_1_ = *d*ns/*s*_s_	0.673	0.5–1.5
*k*_2_	*k*_2_ = *d*nr/*s*_r_	0.714	0.7–1.5
*k*_3_	*k*_3_ = *t*r/*t*_s_	0.75	0.5–1.5
*z*_r_	Number of rotor thread	20	3–20
*z*_s_	Number of stator thread	20	3–20
*L*	Spiral length	160	150–220

**Table 3:** Distribution of sample points and calculation results.

Design Point	*t*_s_ (mm)	*a*_s_ (mm)	*c* (mm)	*k*_1_	*z*_r_	*z*_s_	*k*_2_	*k*_3_	*a*_r_ (mm)	*η* (%)	*H* (m)
1	3.33	1.41	0.20	0.90	12	8	0.71	0.85	2.37	29.33	27.41
2	3.41	0.66	0.25	1.39	19	9	0.89	1.00	1.46	36.65	84.29
3	2.58	0.90	0.40	0.71	11	16	0.82	0.75	2.06	32.01	41.26
4	2.02	1.50	0.43	1.09	16	17	1.20	1.25	1.92	30.24	20.09
5	3.97	0.73	0.26	0.83	12	12	1.07	0.72	0.59	36.14	57.25
6	4.17	1.26	0.30	0.77	15	16	1.69	0.89	0.82	29.36	19.06
7	3.01	1.42	0.24	0.95	16	16	1.04	1.08	0.75	36.36	83.18
8	3.05	1.81	0.41	1.16	15	12	0.92	1.34	0.94	32.36	45.78
9	5.95	1.97	0.20	1.11	9	8	0.76	0.73	2.23	36.00	27.52
…	…	…	…	…	…	…				…	…
283	2.70	1.00	0.26	1.43	16	13	1.76	0.91	1.55	33.47	55.62

**Table 4:** Comparison of optimized design schemes.

Optimizing Parameters	Parameter Symbol and Unit	Original Model	Optimization Results		
			Plan 1	Plan 2	Plan 3
Stator tooth depth	*t*_s_ (mm)	4	3.41	3.01	3.97
Stator tooth thickness	*a*_s_ (mm)	1.7	0.66	1.42	0.73
Radial clearance	*c* (mm)	0.4	0.25	0.24	0.26
Rotor tooth thickness	*a*_r_ (mm)	1.7	1.46	0.75	0.59
*k*_1_	-	0.67	1.39	0.95	0.83
*k*_2_	-	0.71	0.89	1.04	1.07
*k*_3_	-	0.75	1.00	1.08	0.72
Number of rotor heads	*z*_r_	20	19	16	12
Number of stator heads	*z*_s_	20	9	16	12
Pump efficiency	*η* (%)	32.23	35.71	36.36	36.14
Pump head	*H* (m)	70.72	84.29	83.18	57.25

**Table 5:** Comparison of structural parameters of model pumps before and after optimization.

Optimizing Parameters	Parameter Symbol and Unit	Original Model	Optimized Model
Stator tooth depth	*t*_s_ (mm)	4	3.01
Stator tooth thickness	*a*_s_ (mm)	1.7	1.42
Radial clearance	*c* (mm)	0.4	0.24
Rotor tooth depth	*t*_r_ (mm)	3	3.25
Rotor tooth thickness	*a*_r_ (mm)	1.7	0.75
Stator thread lead	*s*_s_ (mm)	120	88.26
Rotor thread lead	*s*_r_ (mm)	112	80.32
Number of stator heads	*z*_r_	20	16
Number of rotor heads	*z*_s_	20	16

**Table 6:** Comparison of target values at the optimal operating point before and after optimization.

Medium Viscosity	Project	Original Model	Optimized Model	Rate of Change (%)
1 cp	*η* (%)	25.94	23.92	−7.77
	*H* (m)	22.39	27.14	21.18
53 cp	*η* (%)	32.23	36.36	12.80
	*H* (m)	70.73	83.18	17.61
133 cp	*η* (%)	39.45	44.79	13.55
	*H* (m)	99.74	119.21	19.53
1200 cp	*η* (%)	44.26	49.35	11.50
	*H* (m)	151.87	168.36	10.85

## Data Availability

Not applicable.

## Nomenclature

*d*_ws_	Outer diameter of stator (mm)
*t*_s_	Stator tooth depth (mm)
*t*_r_	Rotor tooth depth (mm)
*a*_s_	Stator tooth thickness (mm)
*a*_r_	Rotor tooth thickness (mm)
*z*_r_	Number of rotor thread
*z*_r_	Number of stator thread
*s*_s_	Stator thread lead (mm)
*s*_r_	Rotor thread lead (mm)
*c*	Radial clearance (mm)
*L*	Spiral length (mm)
*k*_1_	The ratio of the stator inner diameter to the stator lead
*k*_2_	The ratio of the rotor outer diameter to the rotor lead
*k*_3_	The ratio of the rotor tooth depth to the stator tooth depth
*H*	Pump head (m)
*η*	Pump efficiency (%)
*d*_ns_	Inner diameter of stator (mm)
*d*_nr_	Inner diameter of rotor (mm)
